# Bibliographical Department

**Published:** 1881-02

**Authors:** 


### BIBLIOGRAPHICAL DEPARTMENT.

"Nothing extenuate, nor set down aught in malice."

A PRACTICAL TREATISE ON DISEASES OF WOMEN. By T. Gaillard Thomas, M. D., Prof. of Diseases of Women, in the College of Physicians of New York ; President of the American Gynœcological Society for 1879 ; Vice-President of the New York Academy of Medicine; Surgeon to the New York State Woman's Hospital; President of the Medical Board of the Nursery and Children's Hospital, New York ; Consulting Physician of St. Mary's Hospital for Women, Brooklyn ; Honorary Fellow of the Obstetrical Society of London; Corresponding Fellow of the Obstetrical Society of Berlin ; of the Medical Society of Lima ; and of the Obstetrical Society of Philadelphia; Honorary Member of the South Carolina Medical Association and of the Louisville Medical Society. Fifth edition, enlarged and thoroughly revised ; containing two hundred and sixty-six engravings on wood. Philadelphia. Henry C. Lea's Sons & Co. 1880.

We are indebted to the publishers for a copy of the above admirable work. Indeed, it is a classic production and reflects the highest credit upon its distinguished and learned author. We have been familiar with this work of Professor Thomas for several years, and have always regarded it of the utmost value and importance to the Gynœcologist. In fact, we thought it the best work on the subject in the English language, and were not surprised to learn it had been translated into most of the modern languages of Europe, and used as a text book in the Medical Universities and schools on the Continent. But on comparing this fifth edition with the two preceeding ones in our library, we find that the author has made marked and decided improvements in the text. Reducing where it seemed redundant in the others, adding to and keeping pace with the advances made in Gynœcology; altering and introducing new, and better cuts and illustrations, than those used heretofore, until the work seems now as nearly perfect as is possible of attainment. Dr. Thomas' style and matter are well known to English speaking and reading people, and we will only add that they are unexceptionally good. His continued dedication of the work to Dr. Metcalfe is creditable alike to his head and heart.

The Messrs. Lea are entitled to the highest praise for the superb manner in which they have issued this great work.

Medical Heresies Historically Considered. A Series of Critical Essays on the Origin and Evolution of Sectarian Medicine, embracing a special sketch and review of Homœopathy, past and present. By Gonzales C. Smythe, A. M.. M. D., Professor of the Practice of Medicine, Central College, Indianapolis; Member of American Medical Association, &c.

Philadelphia: Presley Blackiston, 1012 Walnut St.

The Publisher has kindly placed this little volume in our hands and we have read it with pleasure and satisfaction. The author epitomises the various schools and heresies in Medicine in the first 95 pages of the book, and devotes the remainder, 218 pages, to an exposition of the Heresies of Hahnemann. He handles this part of his subject with great intelligence and fairness, and it would be well for the cause of truth, and science, if his unanswerable statements and exposition of the absurdities of that system could be read by those who are disciples of the imposition. He sustains his positions with quotations from the writings of the founder of the absurdities and delusions known as homœopathy at the present day. It would add greatly to the enlightenment of those who adhere to this mythical mode of treatment, if this, and the kindred work of Professor Palmer, could be laid before the public in a popular form. Would not the end to be desired, justify the expenditure of the means necessary for its attainment by the American Medical Association?

The facts and arguments are unanswerable in both those works, and it would seem that somebody, or the profession at large, ought to feel sufficient interest in the cause of humanity to place these impartial expositions of the subject before the reading public! The publisher has done his part neatly and well in issuing the work, and the price places it within the reach of every one who would care to read an exposition of the matter on which it treats.

ROCKY MOUNTAIN HEALTH RESORTS. *An Analytical Study of High Altitudes in relation to the arrest of Chronic Pulmonary Diseases.* By Charles Denison, A. M., M. D., etc., etc. Second Edition.

Boston: Houghton, Miffiin & Co. 1881.

We are indebted to the author for a copy of this work. It is well written and cannot fail to be serviceable to physicians having patients in need of dry air and high altitudes. It furnishes valuable information to such patients themselves in regard to different localities, accomodations, etc.

CHRONIC BRIGHT'S DISEASE IN CHILDREN CAUSED BY MALARIA. By Samuel C. Busey, M. D. In this extract from the transactions of the American Medical Association, Dr. Busey furnishes some interesting facts and experiences.

THE THIRD ANNUAL REPORT OF THE PRESBYTERIAN EYE AND EAR CHARITY HOSPITAL OF BALTIMORE, for the year ending December 1st, 1880. We are indebted to the courtesy of Prof. J. J. Chisolm, M. D., Surgeon in chief, for a copy of this valuable document. The below extract from Dr. Chisolm's report to the Board of Managers will give a better idea of the charity than anything we might say, viz:

The 30th of November closed the 3d years' work of the Presbyterian Eye and Ear Charity Hospital, and a heavy work it has been. When the last year's report was made to the Board of Managers, they concluded, from the very large number of sick treated, that the Institution had attained its fullest growth, and some of the Board thought that very few persons suffering with bad eyes could remain in the city to be cured. We, however, began the first day of the new year by giving professional services and medicines to 92 poor persons, afflicted with painful eyes, ears or throats, and the work has steadily continued, from day to day, until at the end of the year just closed the aggregate visits of the applicants for medical charity reached the enormous number of 23, 368, against 19,683 for last year, and 13,937 for the year previous, an average of 77 persons for each day of the past year. On one bright day 146 persons applied for treatment, and no day has been so cold, rainy or bad, that some were not found awaiting the professional services which the poor suffering from eye disease have learned to value so highly. 21 persons is the smallest number treated on any one day for the past year.

The following have been received from their intelligent and skillful author, viz: *Laparotomy and Colotomy*, with formation of Artificial anus for obstruction of intestines. By William A. Byrd, M. D., Illinois. Extracted from the transactions of the American Medical Association; and *Extirpation of Rectum* without destroying the Sphincter Ani muscle. By the same author. (Re-printed from the *Med. and Surg. Reporter.*) Dr. Byrd has shown much judgment and skill in the performance of his operations, and is undoubtedly one of the most successful Surgeons of his age in the West.

We have received the FIRST ANNUAL REPORT OF THE *Dispensary of Nervous Diseases* from Dr. Van Bibber, attending Physician. The Institution supplies a want in our midst.

*A Spring Course of Anatomy and Operative Surgery* will be delivered at the University of Maryland, beginning on the 1st of March, 1881. By Dr. Randolph Winslow. The fee for the course will be $10. with additional charges of $2.00 for Anatomical material.

An Examination for Three Resident Physicians for the Western Pennsylvania Hospital, to serve for one year, from April 1st, 1881, will be held at the Hospital Building, Pittsburgh, Pa., on the fourth Wednesday of March, 1881, at 10 o'clock, A. M. Competition open to all graduates in Medicine. There are about 1,000 Patients treated annually. Clinical instructions given daily by the staff.

By order of the staff.

Chas. Emmering, M. D., James McCann, M. D., W. J. Asdale, M. D., Examiners.

The following publications have also been received, viz: *The Proceedings of the Louisiana State Medical Association, at its third meeting held in the city of New Orleans, March 31, April 1, and 2d, 1880,* and *Transactions of the Twenty-second annual Meeting of the State Medical Society of North Carolina* Wilmington, N. C., 11th May, 1880.

THE PHYSICIAN'S AND SURGEON'S INVESTIGATOR, a Monthly Journal of Medicine and Surgery, edited by A. A. Hubbell, M. D., has been received and placed with pleasure on our exchange list.

THE MEDICAL ANNALS, A Journal of the Medical Society of the county of Albany, Albany, N. Y., and THE NEW YORK MEDICAL ABSTRACT, a monthly Journal of condensed Medical news, Vol. I, No. 1 have also been received. We wish both of them success.

TRANSACTIONS OF THE AMERICAN DENTAL ASSOCIATION OF THE TWENTIETH ANNUAL SESSION, held at Boston, August, 1880. Through the courtesy of Dr. Geo. H. Cushing, chairman of the publication committee, we are indebted for a copy. The proceedings are of much interest and importance.

TRANSACTIONS OF THE IOWA STATE DENTAL SOCIETY.—Eighteenth Annual Session, held at Oskaloosa, July 13th to 15th, has also been received. It contains instructive articles of value to those interested in the specialty of dentistry.

Caries of the Superior Maxilla, and Carbolic Acid and Creosote, their Chemistry and Therapeutical application to the Practice of Dentistry have been received from Dr. Trumann W. Brophy, their author. Both interesting documents.

Anæsthesia is the title of an interesting paper by W. C. Barrett, M. D., D. D. S., of Buffalo, N. Y., to whom we are indebted for a copy.

Medical Progress, a paper read before the Maryland Homœopathic Medical Society, November 10th, 1880. By Eldridge C. Price, M. D., of this city, contains some interesting facts in regard to the practice of Similia, etc., at the present time. If he would use little larger doses of some preparations he mentions, he would be most decidedly in advance of the founder of his system. A step or two farther, Doctor, read Palmer's and Smythe's expositions and "get out of the wilderness."

The American Medical Bi-Weekly.—This Journal, which was formerly published in Louisville, Ky., and which had nearly reached the close of the eleventh volume when the severe and protracted illness of the Editor compelled its temporary discontinuance, is now, on his entire recovery, restored to its position among the Medical Journals of this country. The Editor, Dr. E. S. Gaillard having made his home in New York, the Bi-Weekly is now published there. The first number of volume twelve has been received. It is now a double column Medical Journal, larger than the old Bi-Weekly, and is published at $1.00 a year; the former terms being $3.00. The Bi-Weekly is with pleasure placed upon the Exchange list. Its address is Box 1124 N. Y. City.

